# Validity and Reproducibility of the Measurements Obtained Using the Flexicurve Instrument to Evaluate the Angles of Thoracic and Lumbar Curvatures of the Spine in the Sagittal Plane

**DOI:** 10.1155/2012/186156

**Published:** 2012-04-24

**Authors:** Tatiana Scheeren de Oliveira, Cláudia Tarragô Candotti, Marcelo La Torre, Patricia Paula Tonin Pelinson, Tássia Silveira Furlanetto, Fernanda Machado Kutchak, Jefferson Fagundes Loss

**Affiliations:** ^1^Physiotherapy Faculty, Universidade do Vale do Rio dos Sinos (UNISINOS), 93022-000 São Leopoldo, RS, Brazil; ^2^Physical Education Department, Universidade Federal do Rio Grande do Sul (UFRGS), 90690-200 Porto Alegre, RS, Brazil

## Abstract

*Objective*. to verify the validity and reproducibility of using the flexicurve to measure the angles of the thoracic and lumbar curvatures. *Method*. 47 subjects were evaluated by: (1) palpation and marking of the spinous processes using lead markers, (2) using X-rays in the sagittal plane to measure the Cobb angles, (3) molding the flexicurve to the spine, and (4) drawing the contour of the flexicurve onto graph paper. The angle of curvature was determined with the flexicurve based on a 3rd order polynomial. *Results*. No differences were found between the Cobb angles and the angles obtained using the flexicurve in thoracic and lumbar curvatures (*P* > 0.05). Correlations were strong and significant for the thoracic (*r* = 0.72, *P* < 0.01) and lumbar (*r* = 0.60, *P* < 0.01) curvatures. Excellent and significant correlations were found for both the intraevaluator and interevaluator measurements. *Conclusion*. The results show that there is no significant difference between the values obtained using the flexicurve and those obtained using the X-ray procedure and that there is a strong correlation between the two methods. This, together with the excellent level of inter- and intraevaluator reproducibility justifies its recommendation for use in clinical practice.

## 1. Introduction

Traditionally, physiotherapy has been considered a good option for both the treatment and prevention of spinal alterations. Postural evaluation is used to identify such alterations or follow the evolution of treatment at the different healthcare levels [1].

Both quantitative and qualitative postural evaluation methods are available to physiotherapists. Without doubt, of the qualitative methods, the most widely used is the plumb line method, which consists in a subjective visual evaluation of parts of the body in relation to its position in space. This method depends on the experience of each evaluator in diagnosing postural deviation and, due to its subjective nature it is difficult to compare diagnoses among physiotherapists [2].

The subjective nature of this traditional method of postural evaluation has encouraged the search for quantitative methods, capable of providing numerical measures of spinal curvature deviation. Since the 1930's, the gold standard method of measuring spinal kyphosis and lordosis has been the X-ray examination [3, 4]. Traditionally, the angles of the spinal curvatures are obtained using the two or four-line Cobb method [4–7]. Despite its confirmed validity, some issues can be raised regarding the use of X-ray examination. First, the patient is exposed to radiation and this therefore is not recommended for periodic examinations [8–11]. Second, an important aspect, particularly in underdeveloped and developing countries is the high cost of the X-ray examination. For example, in Brazil X-ray examinations represent 11.67% of the total spent on moderate complexity examinations [12]. Third, in some countries the physiotherapist is not authorized to request X-ray examinations, this being the exclusive prerogative of the physician.

With this in mind, the use of low-cost, validated, noninvasive instruments may be an alternative to the X-ray examination for the physiotherapist. The use of instruments such as the Moiré topography [13, 14], the spinal pantograph [15], the flexicurve [16, 17], the inclinometer [18], the arcometer [19], the adapted arcometer [20], and the Debrunner's Kyphometer [21] to obtain objective measurements has been widely described in the literature. Among these instruments, the flexicurve stands out due to its capacity to provide a representation of spinal curvature in a continuous line and not only specific points, as with most of the abovementioned instruments. Although Moiré topography and spinal pantography can reproduce the shape of the spine they are not portable, which limits their clinical usefulness.

The flexicurve consists of a flexible metal ruler covered in plastic that can be molded to the back of the individual in order to replicate the shape of the spine. In the literature, some studies have indicated the validity of the flexicurve in protocols involving measurement of the spinal curvature at isolated levels. The flexicurve was validated for use in the lumbar region by Hart and Rose [16], in the cervical region by Harrison et al. [22], and in the thoracic region by Teixeira and Carvalho [17]. To the best of our knowledge, there is a noticeable absence of a single procedure for measuring the thoracic and lumbar curvatures during the same evaluation using o flexicurve, which would facilitate its use both in the clinic and on a large scale in the field. Furthermore, the low cost of the flexicurve together with the ease of use and transport encourages its use in the evaluation of large populations, providing the therapist with a tool that can be used in primary clinical evaluation. In addition, the flexicurve can be a useful tool in field research, particularly for epidemiological research. So, the aims of the present study are to investigate (1) the accuracy of the angles of thoracic and lumbar curvatures of the spine obtained in the sagittal plane using the flexicurve instrument by comparing them with those obtained using X-ray exams and (2) the inter- and intraevaluator reproducibility of the flexicurve.

## 2. Methodology

The sample consisted of 47 subjects of both sexes with an average age of 44.9 (±19.4), body mass of 77.6 (±16.3) kg, height 1.68 (±0.09) m, and body mass index of 27.5 (±5.0). The inclusion criteria were the prescription for X-ray examination of the spine or thorax, and the exclusion criteria were the presence of spina bifida, six lumbar vertebrae, diseases or disabilities that affect the orthostatism, and unclear X-ray images. The study was approved by the Research Ethics Committee, and all the subjects signed the free informed consent (FIC) form, and all the procedures used were in accordance with Helsinki declaration.

In order to determine whether the flexicurve constitutes a valid and reliable method, three sets of information were collected: (1) the veracity of the angles of thoracic and lumbar curvatures of the spine obtained in the sagittal plane by comparing them with those obtained using X-ray exams (*n* = 47); (2) the interevaluator reproducibility of the instrument based on the measurements taken by three different evaluators, in the same place, on the same day (*n* = 15); (3) the intraevaluator reproducibility of the instrument based on a comparison of the measurements obtained with the use of the flexicurve by the same evaluator on two different occasions at a one-week interval (*n* = 15).

Firstly, in the waiting room of the clinic, patients were invited to participate in the study, the nature of which was explained and they were asked to sign the FIC. After this, the subjects were led to the X-ray room, where three procedures were carried out on the same day: (1) anthropometric and identification data were obtained; (2) X-ray exam; (3) evaluation with the flexicurve instrument.

**Figure 1:**
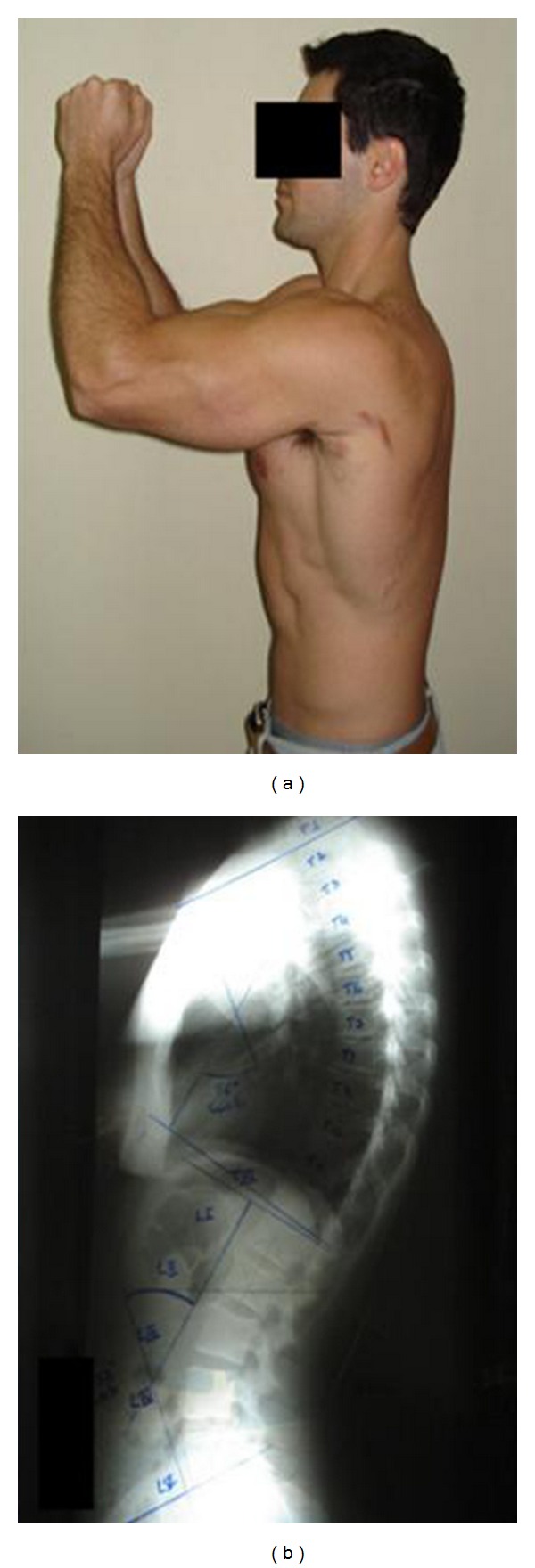
(a) Position of the subjects during both evaluation procedures; (b) X-ray image of the spine.

For the X-ray exam and flexicurve evaluation, each subject stood barefoot with the back uncovered while the spinal processes of the C7, T1, T12, L5, and S1 vertebrae were identified and marked. The spinous processes were identified using palpation and marked by only one of the evaluators. In order to confirm the correct identification of the vertebrae of interest, small lead pellets were taped to the skin so that could be seen in the X-ray image. It should be pointed out that during “normal” use of the flexicurve there is no need to use lead pellets. The C7, T1, T12, L5, and S1 spinal processes can be marked using a demographic pencil. During both procedures, the subjects were instructed to remain standing with the knees straight, feet parallel and the shoulders and elbows at 90° of flexion (Figure 1(a)). This position was adopted in order to avoid the humerus appearing in front of the spinal column (Figure 1(b)) [23, 24].

The X-ray exams were performed by a radiologist using a device made by *Siemens*, X-ray film from *Fuji Films, *and a processor from *Kodak*. During the X-ray exam, each subject was in apnea with the leftside closest to the X-ray source. For the thoracic spine the focus was maintained on the seventh costal arch, while for the lumbar spine the focus was maintained 3 cm above the anterior-superior iliac spinous. The settings for the X-ray machine for thoracic spine were minimum focus: 200; mAs: 0.7; kVp: 78; maximum focus: 200; mAs: 0.8; kVp: 88. For the lumbar spine were: minimum focus: 200; mAs: 0.7; kVp: 78; maximum focus: 300; mAs: 1.0; kVp: 105.

**Figure 2:**
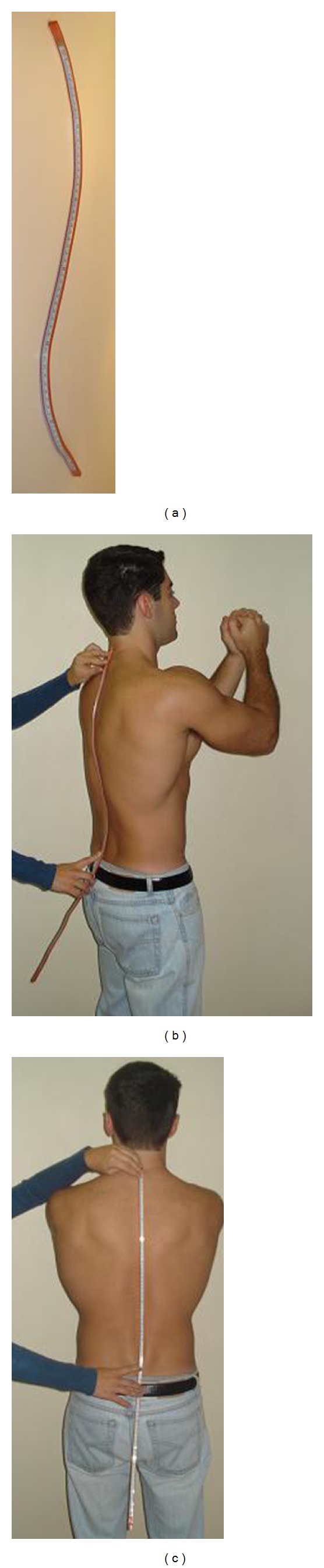
(a) Flexicurve; (b) molding the flexicurve to the spine, lateral view; (c) molding the flexicurve to the spine, posterior view.

Immediately following the X-ray, the lead pellets were removed and while the subject remained in the same position an evaluation of the spinal curvature was made using the flexicurve. The flexicurve (*Trident*) is a flexible plastic-covered metal ruler, 80 cm in length, marked at 1 mm intervals. This instrument can be molded to rounded structures (Figure 2(a)). The assessment procedure with the flexicurve consisted of molding the instrument to the shape of the spine from the C7 to the S1spinal processes (Figures 2(b) and 2(c)).

The measurements with the flexicurve were taken by three evaluators (Eva1, Eva2, and Eva3), each of which was blind to the measurements obtained by the other, to determine the degree of agreement of the measurements obtained by the evaluators (interevaluator comparison). This procedure was adopted to avoid a bias evaluation.

**Figure 3:**
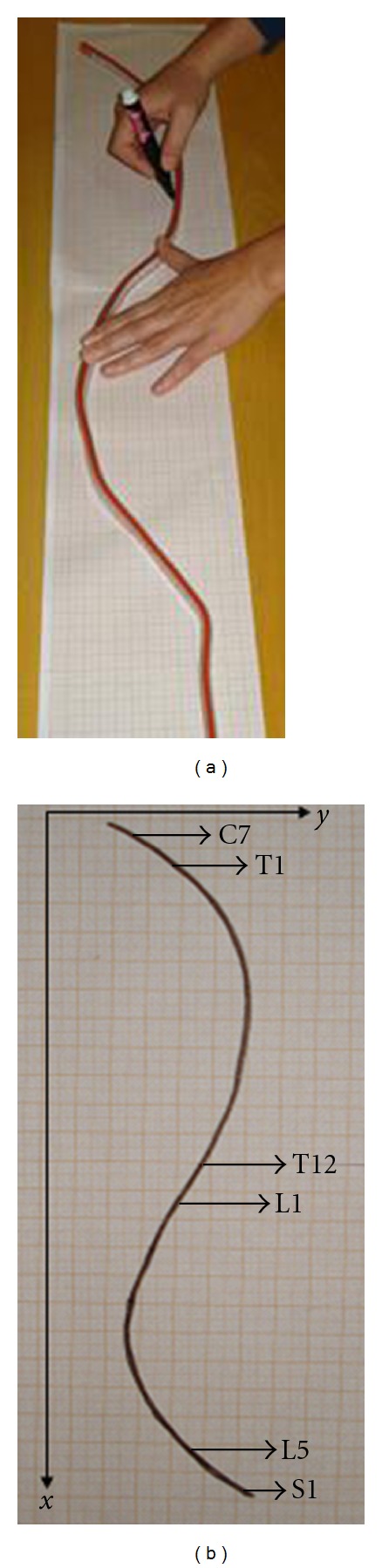
(a) Tracing the internal contour of the flexicurve; (b) representation of the thoracic and lumbar curvatures on graph paper showing the position of the spinous processes.

Although the spinous processes (C7, T1, T12, L1, L5, and S1) were identified using palpation by only one of the evaluators, all the three evaluators performed the following steps when using the flexicurve to assess spinal curvature. While molding the flexicurve to the contour of the spine, the C7, T1, T12, L1, L5, and S1 spinal process were located and recorded using the metric scale incorporated in the instrument. After molding the contour of the spine the flexicurve was removed and the internal edge (the side of the flexicurve in contact with the skin) was traced onto graph paper (Figure 3(a)), thus representing the thoracic and lumbar curvature in the sagittal plane, with the spinous process of interest identified (Figure 3(b)). 

**Figure 4:**
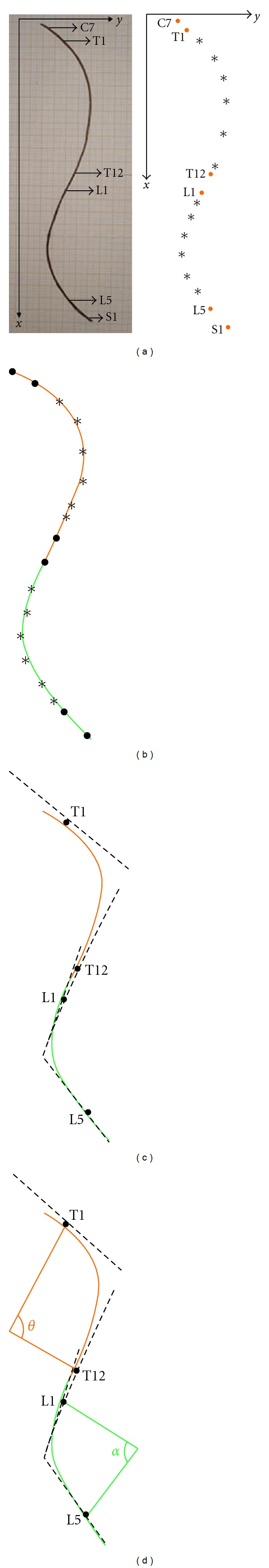
(a) Outline on graph paper of the spine and points representing the shape of the lumbar and thoracic curvatures; (b) drawing of the curvatures obtained using two 3rd polynomial; (c) drawing the tangents on the limit points of the curvatures (T1/T12 for thoracic, L1/L5 for lumbar); (d) drawing the straight lines perpendicular to the tangents and establishing the thoracic (*θ*) and lumbar (*α*) angles.

A Cartesian coordinate system was defined on the graph paper where the *x*-axis represents the cranial-caudal direction, and the *y*-axis represents the anterior-posterior direction (Figure 3(b)). Using the coordinate system 18 paired coordinates (*x*, *y*) were marked on the curve (Figure 4(a)). Based on these 18 pairs of coordinates, two sets of 10 pairs of coordinates were selected (two pairs were common to both sets), one for each level of the spine (thoracic and lumbar). For the thoracic curvature, the 1st pair of coordinates corresponded to the location of C7, the 2nd to T1, the 9th to T12, and the 10th to L1. For the lumbar curvature, the 1st pair of coordinates corresponded to the location of T12, the 2nd to L1, the 9th to L5, and the 10th to S1. The remaining pairs of coordinates were marked equidistantly with the naked eye between T1 and T12 for the thoracic spine and between L1 and L5 for lumbar spine (Figure 4(a)). These procedures were carried out by the three evaluators under the same conditions and using the same instrument in order to verify the interevaluator reproducibility of the instrument.

The coordinates representing the thoracic curvature (ten pairs) and the lumbar curvature (another ten pairs) were introduced in an algorithm developed in Matlab software. The algorithm produce the angles of curvature, occurring between the points representing the spinous process of T1 to T12 for the thoracic curvature and from L1 to L5 for the lumbar curvature, based on the following procedures (Figure 4).

1. Two 3rd order polynomial were fitted representing, respectively, the shape of the thoracic curve (1st to 10th coordinates, C7 and L1) and lumbar curve (1st to 10th coordinates, T12 and S1) of the subject under assessment (Figure 4(b)).
2. The 1st derivative of the fitted 3rd order polynomial was calculated. This procedure provides the equation representing the family of tangents to the 3rd order polynomial. This equation was used to extract the inclination of the tangents to the points T1 and T12 (thoracic) and L1 and L5 (lumbar).
3. With the inclination of the tangents and the coordinates of each end point (T1, T12, L1, and L5) it was possible to obtain the equations corresponding to the tangents of these points (Figure 4(c)).
4. Based on the tangents the equations of the perpendicular lines crossing the meeting points T1, T12, L1, and L5 were calculated.
5. The angle of the flexicurve (FA) was calculated considering the intersection of the perpendicular straight lines, named as *θ* for the thoracic curvature (FA_T_) and *α* for the lumbar curvature (FA_L_) (Figure 4(d)).

The subjects who agreed to participate in the second evaluation day were required to return to the same place at the same time one week later, in order to be reevaluated by one of the three evaluators. On this second day only the flexicurve was used for the evaluation. This procedure made it possible to determine the degree of agreement between the measurements obtained on two different days by the same evaluator (intraevaluator comparison).

The two-line Cobb method was use to obtain the angles of the curvatures of the thoracic and lumbar spine in the based on the X-ray exam taken from the sagittal plane. This method consist of tracing two straight lines, one extending from the top edge of the upper vertebra and the other extending from the bottom edge of the lower vertebra, respecting the inclination of the vertebrae. The Cobb angle (CA) is formed where these lines meet [25, 26]. This procedure was followed in order to identify the Cobb angles for the thoracic spine (CA_T_) based on T1 and T12, and for the lumbar spine (CA_L_) based on L1 and L5. The angles obtained from the X-ray exam were used as the “gold standard” in the evaluation of the proposed method.

The angles obtained with the flexicurve (FA_T_ and FA_L_) and from the X-ray exams (CA_T_ and CA_L_) were submitted to inferential statistical treatment using version 15 SPSS software. Initially the normality and homogeneity of the data for both curvatures were confirmed using the Kolmogorov-Smirnov and Levene tests, respectively. One-way ANOVA for repeated measurements showed there was no difference between the FA obtained by the three evaluators in either the thoracic (FA_T_) or lumbar curvatures (FA_L_). Therefore, for the purpose of comparing FA_T_ × CA_T_ and FA_L_ × CA_L_, only the values obtained by evaluator 1 were used. The Pearson Correlation and Student's *t*-(for paired samples) tests were used to verify relationship between the angles of the curvatures obtained using the flexicurve and X-ray exam. The classifications for the Pearson Correlation Coefficients were strong (>0.5); medium (form 0.3 to 0.5); small (from 0.1 to 0.3); none (<0.1) [27]. Bland and Altman's graphic analysis technique was used to verify the agreement between the FA_T_ × CA_T_ and FA_L_ × CA_L_ [28, 29]. In order to assess the intra- and interevaluator reproducibility of the angles of curvature obtained using the flexicurve, the Intraclass Correlation Coefficient (ICC) was used (*α* < 0.05). The ICC results were classified as excellent reliability (ICC > 0.75); good reliability (ICC from 0.40 to 0.75); poor reliability (ICC < 0.40) [30].

## 3. Results

When the measurements obtained by the three different evaluators were compared no significant differences were found between them in either the thoracic curvature *F*(1.56, 21.94) = 2.830, *P* > 0.05, or the lumbar curvature, *F*(1.40, 19.72) = 2.478, *P* > 0.05. The ICC values obtained with the flexicurve showed excellent reproducibility between the evaluators with values of 0.94 for thoracic and 0.83 for lumbar curvature (Table 1). Likewise, the results for intraevaluator, based on the results obtained by evaluator 1 on different days, showed excellent reproducibility, with ICC values of 0.83 for the thoracic and 0.78 for the lumbar curvature. These results made it possible to compare the X-ray with the flexicurve using only the results obtained by evaluator 1, which are summarized in Table 2. The mean CA_T_ (*M* = 43.7, SE = 1.6) values were similar to those of the FA_T_ (*M* = 42.9, SE = 1.3, *t*(46) = 0.723, *P* = 0.473). Likewise, the mean CA_L_ (*M* = 40.5, SE = 1.5) values were similar to those of the FA_L_ (*M* = 40.0, SE = 1.2, *t*(46) = 0.416, *P* = 0.679).

The mean values, standard deviations, and range were very similar for both measuring techniques in both curvatures. In the thoracic curvature, more than half the individuals (55.3%) showed a difference of less than 5° and 80.9% less than 10° between the techniques. There is a good relationship between the CA_T_ e FA_T_ values as demonstrated by the small relative (0.8°) and absolute (6.5) mean differences. The results of the Pearson's Correlation test, between CA_T_ and FA_T_, showed a strong and significant correlation for the thoracic curvature (*r* = 0.70; *P* = 0.01). In the lumbar curvature, almost half the subjects (48.9%) showed a difference of less than 5°, and 76.6% less than 10° between the techniques. There is a good relationship between the CA_L_ e FA_L_ values as demonstrated by the small relative (0.5°) and absolute (6.8) mean differences. The results of the Pearson's Correlation test, between CA_L_ and FA_L_, showed a strong and significant correlation for the lumbar curvature (*r* = 0.60; *P* = 0.01).

**Figure 5:**
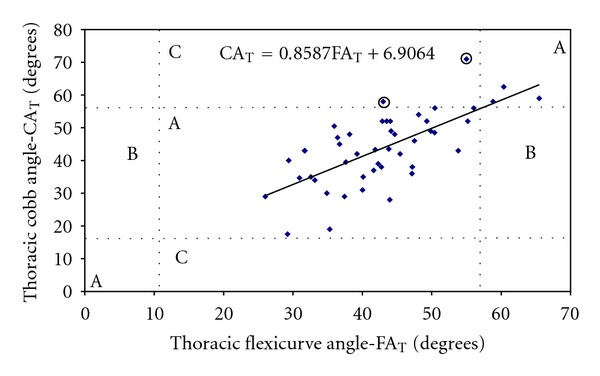
The distribution of the flexicurve angle in relation to the Cobb angle. The vertical and horizontal dotted lines represent the distribution of a normal curvature [31] and divide the figure in 9 zones. The same results are obtained with both techniques in the “A” zones, false positives should appear in “B” zones; false negatives (⊙) are shown in the “C” zones.

The regression analyses also indicate a good relationship between both methods. In Figure 5, the normal range for thoracic curvature, extracted from Bernhardt & Bridwell [31], was marked with dotted lines. Horizontal dotted lines were drawn at 16° and 56° for the Cobb angle (CA_T_) and vertical dotted lines at 11° and 57° for the Flexicurve angle (FA_T_). The measurements obtained with the two methods do not agree in only two of the subjects, and they are marked with a circle in the graph.

**Figure 6:**
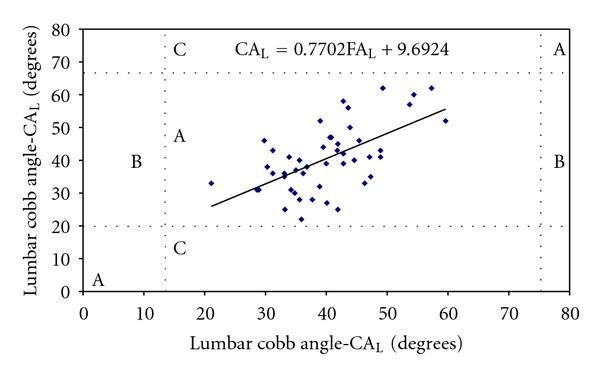
The distribution of the flexicurve angle in relation to the Cobb angle. The vertical and horizontal dotted lines represent the distribution of a normal curvature [31] and divide the figure in 9 zones. The same results are obtained with both techniques in the “A” zones, false positives should appear in “B” zones; false negatives should appear in the “C” zones.

In Figure 6, the normal range for lumbar curvature, extracted from Bernhardt & Bridwell [31], was marked with dotted lines. Horizontal dotted lines were drawn at 20° and 68° for the Cobb angle (CA_T_) and vertical dotted lines at 13° and 76° for the Flexicurve angle (FA_T_). All individuals were classified as “normal” using both techniques.

**Figure 7:**
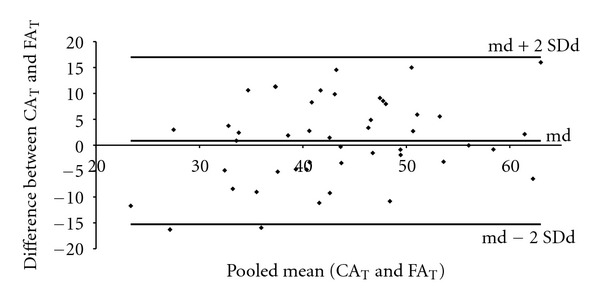
Graphic representation of the degree of agreement in relation to the difference between the CA_T_ and FA_T_ in function of the pooled mean (CA_T_ and FA_T_). The thoracic curvature mean of differences (md) = 0.8°, the Standard Deviation of difference (SDd) = 8.0°, and the limits of agreement are from −15.3 to +17.0°.

**Figure 8:**
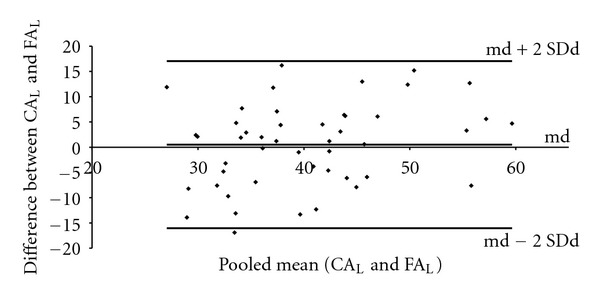
Graphical representation of the degree of agreement in relation to the difference between CA_L_ and FA_L_ in function of the pooled mean (CA_L_ and FA_L_). The lumbar curvature mean of differences (md) = 0.5°, the Standard Deviation of difference (SDd) = 8.3°, and the limits of agreement are from −16.0 to +17.0°.

Figures 7 and 8 show the Bland and Altman graphic analysis procedure for the thoracic and lumbar curvatures, respectively. The mean of the differences between the CA and the FA was 0.8° for the thoracic curvature and 0.5° for the lumbar curvature. Figures 7 and 8 show that most of the points representing the difference between the CA and FA values are within the limits of agreement (md ±2 SDd), with random dispersion and average differences approximate to zero.

## 4. Discussion

The aim of the study was to validate the flexicurve instrument for measuring the thoracic and lumbar angles of curvature by comparing measurements obtained using flexicurve with those obtained using X-ray exams. The results show that (1) no significant difference was found between the angles obtained with each method in either curvature; (2) a strong and significant correlation was found between the two methods; (3) there was agreement between the angles obtained using the two methods. These results indicate that the flexicurve can be considered valid for use in the evaluation of the thoracic and lumbar curvatures in the spinal column. Moreover, in general the results obtained demonstrate that the flexicurve instrument have excellent levels of intra- and interevaluator reproducibility, for both the thoracic and lumbar curvatures.

In the literature the flexicurve has been seen as an alternative instrument capable of providing information about spinal curvature. Accordingly, some studies have indicated the reproducibility [16, 17, 32, 33] and validity [16, 17, 34] of using the flexicurve in the thoracic spine [17] or in the lumbar spine [16, 34]. No studies were found that demonstrate the agreement of the results obtained using the flexicurve in relation to the gold standard, only the relationship of flexicurve to the gold standard. The main difference and great innovation contained in the proposed method, in relation to earlier studies, is the possibility of simultaneously measuring the thoracic and lumbar curvatures. Furthermore, the vast majority of these methods does not allow simultaneous evaluation of the thoracic and lumbar curvatures in the same collection procedure and only shows the results of the correlation, while failing to follow graphical statistical evaluation procedures for the agreement. Considering the information on the flexicurve found in the literature to date, the purpose of this study is to identify the advantages and limitations of the instrument so that it might be more widely used in clinical practice with a reasonable degree of confidence.

In relation to the evaluation of reproducibility of the flexicurve in the thoracic curvature and lumbar curvature, Teixeira and Carvalho [17] and Lovell et al. [32] reported an interevaluator ICC of 0.94 and 0.50, respectively, obtained by two evaluators. In the present study interevaluator ICC values of 0.94 and 0.83 were found for the thoracic curvature and lumbar curvature (Table 1), thus demonstrating that the flexicurve method has excellent level of interevaluator reproducibility. However the assessment was based on the results obtained by three evaluators rather than two.

Furthermore, Teixeira and Carvalho [17] when assessing the intraevaluator reproducibility of the flexicurve for the thoracic curvature obtained an ICC of 0.87, which is close to value found in the present study, 0.82. Regarding the results of the intraevaluator reproducibility of the flexicurve in the lumbar curvature, the present study obtained an ICC of 0.78. Hart and Rose [16] and Walker et al. [33] reported an ICC of 0.97 and 0.90, respectively, between intra-evaluator measurements obtained with the flexicurve for the lumbar curvatures. Although the value obtained by these authors is higher than that found in the present study the measurements were made during the same session on the same day, while in the present study the measurements were made on two distinct days, at an interval of one week. It can therefore be suggested that these differences in methodology may have contributed towards the differences between the assessments of intraevaluator reproducibility found in the three studies.

In relation to the evaluation to validity of the flexicurve in the thoracic curvature, Teixeira and Carvalho [17] compared angles from 56 individuals, obtained using the Cobb method from X-rays with those obtained from the same individuals using the flexicurve. Their results demonstrated a significant correlation (*r* = 0.86) between the measurements and a mean difference between methods of 0.9°, which is higher than that found in this study. However, the reliability of the method proposed by Teixeira and Carvalho [17] can be questioned because they did not provide the results of comparison between the methods nor any assessment of the agreement between the methods. In relation to the evaluation to validity of the flexicurve in the lumbar curvature, Hart and Rose [16], found a correlation (*r* = 0.87) between the angles obtained with the flexicurve and the X-ray. However, they did not show mean values for the comparison of the angles nor the agreement between the methods and used a very limited sample (*n* = 6).

 The relative mean differences between the flexicurve and radiologic data are small (<1°) for both the thoracic and lumbar curvatures (Table 1). Although, the range of values is quite wide (±16°), the symmetrical distribution of the values for both curvatures suggests a random error. In fact, the subjects who had a difference greater than 10° did not belong to a specific class or have different Cobb angle amplitude (Table 1). The range of curvatures is comparable with the values obtained in previous studies [15, 19, 20, 24, 31]. However, methodological factors, such as the improper handling of the instrument leading to a loss of the spinal curvature format during the transfer of the format to the paper, could explain the greater discrepancies seen in specific cases. Other factors might be related to the morphology of the individuals such as increased adiposity.

 A wide variety of instruments and methods of evaluating spinal curvature are reported in the literature [15–17, 20]. Whatever the tool used to evaluate the spine, there is always the possibility of errors inherent in any method. Even with X-ray examination, which is considered the gold standard, an error of approximately ±5° has been accepted for the Cobb angle [35]. It is for the therapist to choose from among the various methods found in the literature, the one with less chance of error. In the case of the flexicurve, measurement error may be associated with (1) patient positioning, (2) surface palpation of spinal processes, (3) modeling and placement of flexicurve tape, (4) loss of format of the flexicurve and its alignment on the paper/coordinates system, and (5) while marking the pairs of coordinates. Nevertheless, palpation in order to identify the correct location of anatomical landmarks is an essential prerequisite to ensure the reliability and reproducibility of postural analysis [36], and the spine is one of the regions of the body that provides the greatest difficulty for the examiner, due to several factors, such as the large number of spinal segments, most of which are relatively small, only the spinous processes are relatively close to the skin surface and the high degree of variation in the shape and orientation of the spine [37, 38]. Therefore, it is recommended that this phase of the evaluation with the flexicurve always be performed by an experienced assessor, since it has been demonstrated that the degree of clinical experience interferes with the quality of the palpation [39].

 When the use of an alternative instrument for obtaining spinal measurements is suggested, some degree of reliability of the measurements is expected. This is not a simple task, given the complexity of the spine. The results of the present study demonstrate that, at least as regards the classification of subjects within the range of normality, there seems to be a degree of certainty. Only two false-negative results were found in the thoracic curvature (Figure 5), while all the measurements agreed in the lumbar curvature (Figure 6). It is worth mentioning the wide range that is accepted as normal in the literature [31] and the relatively narrow range of curvatures evaluated in this study. Nevertheless, the combination of results with high intraevaluator reproducibility (Table 1) suggests that any errors, even if marked (>10°), were associated with peculiarities of the subject assessed. From a practical standpoint, Figures 5 and 6 can aid the therapist when deciding which procedure to adopt with the patient. For example, subjects evaluated with the flexicurve as not having angles within the range of normality should be advised to undergo panoramic X-ray examination of the spine in the sagittal plane, to confirm the diagnosis.

 The use of correlation and regression analyses reflects the relationship between both approaches but not the agreement between them [19, 24]. Here, the graphic analysis technique, proposed by Bland and Altman [28] was used to evaluate the degree of agreement between the FA and CA. The technique consists of plotting the difference between the measurements obtained using the two methods (CA-FA) in relation to the pooled mean of the same measurements. This procedure provides a view of the pattern of the degree of agreement between the instruments within a range of variation of designated proportions. The random dispersion of the differences, regardless of the magnitude of the measured angles, reinforces the other results of this study, suggesting that the differences are random and not associated with a specific range of the angle of curvature (Figures 7 and 8).

In summary, this study differs from the others because it (1) provides all the necessary requirements for a validation process, which are the result of correlation, comparison, and agreement of the indirect method with the “gold standard” and the inter- and intraevaluator reproducibility and (2) provides a single collection procedure, which simultaneously provides the angular values of the thoracic and lumbar curvatures, thus facilitating the process of assessing the sagittal curvatures of the spine.

Hence, given the validity and reproducibility demonstrated in the present study and considering the low-cost, portability, and the noninvasive nature of the flexicurve instrument it will be useful to provide physiotherapists with some guidelines for its use. First, the angles obtained with the flexicurve need to be corrected using linear transformation. Figures 5 and 6 provide the linear equation that is used to approximate the flexicurve angles to those of the Cobb angles. For example, if the measurement of the thoracic curvature obtained using the flexicurve results in a value of 40°, this number should be multiplied by 0.8587 and the partial result added to 6.9°, resulting in a final value of 41.2°. Second, the instrument is appropriate for primary evaluation of large populations, providing information that can be used in the formulation of public health policies, as well as guiding the conduct of physiotherapists in the field of collective health. Finally, the excellent results obtained regarding inter- and intraevaluator reproducibility indicate that the flexicurve can be used in clinical evaluation to monitor treatment progress.

## 5. Conclusion

 The results of this study demonstrate that there is no significant difference between the values obtained using the flexicurve and those obtained using the X-ray procedure, and that there is a strong correlation between the two methods. Thus the flexicurve can be used to obtain excellent reproducible measurements (inter- and intraevaluators) in the thoracic and lumbar curvature of the spine in the sagittal plane in a single procedure during the same evaluation, making it easy for healthcare professionals to assess posture in all levels of health care.

## Figures and Tables

**Table 1:** Intraclass Correlation Coefficient (ICC) between the evaluations of the thoracic and lumbar curvatures measured with the flexicurve performed by three different evaluators (Eva1, Eva2, and Eva3) and between the first and second evaluation days performed by the same evaluator (day1, day2).

		95% Confidence interval		
	ICC	Inferior limit	Superior limit	*P*
Thoracic (Eva1, Eva2, and Eva3)	0.942	0.865	0.979	<0.001
Lumbar (Eva1, Eva2, and Eva3)	0.831	0.604	0.938	<0.001
Eva1 (day1) × Eva1 (day2): Thoracic	0.829	0.565	0.939	<0.001
Eva1 (day1) × Eva1 (day2): Lumbar	0.783	0.468	0.922	<0.001

**Table 2:** X-ray and flexicurve measurements of the thoracic and lumbar curvature.

Curvature	X-ray mean (SD) range	Flexicurve mean (SD) range	Difference mean (SD) range	Absolute difference mean (SD)	≤5° *n*	5° < *x* ≤ 10° *N*	>10° *N*
Thoracic	43.7 (11.0)	42.9 (8.8)	0.8 (8.1)	6.5 (4.7)	26	12	9
	18° to 71°	26° to 65°	−16° to 16°				
Lumbar	40.5 (10.1)	40.0 (7.9)	0.5 (8.3)	6.8 (4.6)	23	13	11
	22° to 62°	21° to 59°	−17° to 16°				
